# Community-led HIV self-testing for men who have sex with men in Lebanon: lessons learned and impact of COVID-19

**DOI:** 10.1186/s12961-021-00709-x

**Published:** 2021-04-21

**Authors:** Ismael Maatouk, Mostafa El Nakib, Moubadda Assi, Patrick Farah, Bertho Makso, Clara El Nakib, Alissar Rady

**Affiliations:** 1 Dermatology Department, Clemenceau Medical Center Affiliated With Johns Hopkins, Beirut, Lebanon; 2 National AIDS Program, Beirut, Lebanon; 3 Soins Infirmiers et Développement Communautaire (SIDC) Community Center, Mount Lebanon, Lebanon; 4 Proud Lebanon’s Community Center, Beirut, Lebanon; 5 Faculty of Medicine, American University of Beirut, Beirut, Lebanon; 6 World Health Organization Country Office, Beirut, Lebanon

**Keywords:** HIV, Self-test, Testing, Diagnosis, COVID-19, Eastern Mediterranean Region

## Abstract

**Background:**

In Lebanon, HIV is concentrated in both native and refugee communities of men who have sex with men (MSM). For over 10 years, the National AIDS Program (NAP) has offered HIV voluntary counselling and testing through a partnership with nongovernmental organizations (NGOs). In 2018, implementation of HIV self-tests (HIVST) was introduced, and this self-care intervention has been further scaled up during the coronavirus disease 2019 (COVID-19) pandemic. This paper (1) describes the effectiveness of implementing HIVST in Lebanon, and (2) discusses how the success of HIVST implementation has been reflected during the COVID-19 pandemic.

**Methods:**

The NAP conducted a series of workshops (July–November 2018) to introduce HIVST services for healthcare workers working at different NGOs. The workshops highlighted that HIVST would be distributed for free, that it would be confidential and voluntary, and that participants were encouraged to notify the NGOs of their results, which would be kept strictly confidential. NGOs collected data anonymously and confidentially from beneficiaries (age, consistency of condom use and HIV testing history), who were asked to call back with the results of their HIVST. At the NAP, data were combined, aggregated and analysed.

**Results:**

In 2019, the NGOs distributed 1103/1380 (79.9%) HIVST kits to their beneficiaries. The NGOs collected feedback on 111 kit results, of which two were HIV-positive. Feedback about HIVST results from beneficiaries was low (111/1103) due to noncompliance of beneficiaries and the lack of human and financial resources in the NGOs. From January through May 2020, a total of 625/780 HIVST kits (80.1%) were distributed. This period was divided into pre-COVID-19 and during COVID-19. The follow-up with the beneficiaries during COVID-19 was much improved because of the absence of on-site activities, shifting more efforts towards HIVST (449/625). There have been no reports of social harm related to HIVST.

**Conclusion:**

HIVST implementation in Lebanon serves as an example of introducing a self-care intervention as part of a community-led effort. In order to maintain HIVST services at the same improved level, reorganization of care is needed within each NGO following the adaptation process due to COVID-19, along with continuous monitoring and evaluation of HIVST reported data.

## Background

According to the National AIDS Program (NAP), there were an estimated 2570 people living with HIV (PLHIV) in Lebanon in 2019, and the HIV epidemic was concentrated in men who have sex with men (MSM) [1].

Sexual transmission risks among MSM are significantly shaped by inequitable social and structural contexts that influence individuals’ sexual practices and access to HIV prevention. Individual-level acquisition risks include lack of condom use (42.7%), multiple sex partners per month (3 to 4), sex work (27.9%) and lack of HIV testing (69.7%) [1]. Social contexts include habits and norms regarding sexual orientation and/or HIV-positive serostatus, including among families, friends, and cultural, religious and health institutions, which lead to the exclusion of MSM in HIV prevention, education and employment, and in healthier long-term relationships [2]. This has been supported by previous papers on Lebanese MSM, where men in relationships were much more likely to have been tested for HIV [3]. Structural contexts such as the criminalization of homosexuality and the healthcare system also shape the HIV response. Additionally, Lebanon has a community of migrant and refugee MSM who are considered even more vulnerable because they may lack or avoid access to the healthcare system due to stigma and discrimination by providers [4]. Individual-level acquisition risks in migrant MSM include low rates of condom use (36.5%), multiple sex partners per month (3 to 4), sex work (35%) and low HIV testing rates (48.3%) [1]. In local and foreign MSM communities, HIV prevalence was 12% and 3%, respectively, and knowledge of key HIV-related information (testing, prevention, treatment and services) was 8.7% and 6.3%, respectively [1].

For more than 10 years, the NAP has adopted free voluntary counselling and testing (VCT) for HIV. Rapid diagnostic tools are procured by the NAP and provided to MSM, refugees and those who need them, and distributed by nongovernmental organizations (NGOs) and primary healthcare centers (PHCs). The NGOs that provide free HIV testing services (defined as “HIV thematic NGOs”) have a long-term partnership with the NAP. Healthcare workers (HCWs) who work at these NGOs are regularly trained by the NAP on risk assessment, counselling, diagnostic tools, HIV knowledge, stigma and discrimination, associated harm, and NAP reporting tools. Following these trainings, HCWs (who are frequently but not exclusively from key communities, offering services to their peers) deliver VCT services in NGOs and PHCs, confidentially and anonymously, both on-site and through outreach activities. Moreover, NGOs distribute condoms and information, education and communication (IEC) material for free, which is all procured by the NAP. The NGOs send a monthly report to the NAP with the number of tests conducted through the VCT services and the number of positive and negative cases. People who test positive are provided a list of physicians who confirm, assess and prescribe antiretroviral therapy (ART) which is provided for free by the NAP ART dispensing center. According to the NAP, VCT is the most frequently reported diagnostic screening tool, compared to more standard laboratory testing, among MSM because it offers free and anonymous counselling and HIV testing, which provides a sense of security, less stigma and more friendly environments towards their sexual orientation.

In 2014, the Joint United Nations Programme on HIV/AIDS (UNAIDS) released *90–90–90: An ambitious treatment target to help end the AIDS epidemic*, setting three global targets to be achieved by 2020: 90% of all people living with HIV would know their HIV status; 90% of people with diagnosed HIV infection would receive sustained ART; and 90% of all people receiving ART would have viral suppression [5]. Additionally, to scale up HIV testing, WHO published the first global guidelines on HIV self-testing (HIVST) in 2016 [6]. HIVST is a process whereby a person who wants to know their HIV status collects a saliva/blood specimen, performs a test and interprets the result in private. HIVST is a screening test; it does not provide a diagnosis, and confirmatory testing is required if the initial result is positive [6]. HIV antibodies can be detected by HIVST following a “window period” of 1 to 3 months after exposure. HIVST was then endorsed by the WHO Eastern Mediterranean Region [7, 8]. In line with these recommendations, the NAP adopted HIVST in the second half of 2018 to fill the gap between the first (94%) and the second (69%) 90s of the UNAIDS 90–90–90 target. This strategy aimed to reach the largest possible number of PLHIV who remained undiagnosed.

HIVST is an example of a self-care intervention. In 2019, WHO published its first guideline on self-care interventions for health, with a focus on sexual and reproductive health and rights. WHO defines self-care as *the ability of individuals, families and communities to promote health, prevent disease, maintain health, and to cope with illness and disability with or without the support of a health worker* [9]. The concept of self-care recognizes individuals as active agents in managing their own health, including disease prevention and self-medication, and providing care to dependent persons. Self-care interventions for health, such as drugs, devices, diagnostics and/or digital products, can be used with or without the direct supervision of healthcare personnel. Additionally, quality self-care interventions should be accessible and available to everyone, and especially vulnerable populations [10].

Since the beginning of the coronavirus disease 2019 (COVID-19) pandemic, HIV testing has become a significant challenge, and increased efforts were needed to augment access to and facilitate testing [11]. In Lebanon, due to COVID-19 restrictions (mid-March to the beginning of June 2020), HIVST became the only diagnostic tool that could be easily used by people who wished to get tested. This is particularly true for Lebanon since the beginning of COVID-19 (February 2020). During the lockdown, all NGOs were required to stop all on-site services, including VCT, and HIVST became the only available option for beneficiaries needing an HIV test.

This paper has two objectives: (1) to describe the effectiveness of implementing HIVST in Lebanon (adoption of HIVST) and (2) to discuss how the success of HIVST implementation has been impacted specifically through a comparison of services pre-COVID-19 and during COVID-19 (adaptation of HIVST services).

## Methods

In mid-2018, the NAP conducted a series of workshops to introduce HIVST services, which included presenting the scientific evidence of the test, providing IEC material and HIVST hands-on support to different HCWs working in NGOs. A total of 280 HCWs and volunteers from different NGOs were reached by the workshops. The workshops’ key messages about HIVST were that (1) tests are distributed for free to the beneficiaries; (2) tests provide more privacy since beneficiaries do not have to be physically in front of a counsellor during the test; (3) tests are voluntary; (4) test results are not shared directly with the NGOs, but beneficiaries should be encouraged to notify the NGOs about their test results, without any breach of confidentiality; and (5) tests should target frequent testers and people who avoid classic VCT services because of fear or stigma.

Following the workshops, several educational and informative resources were produced and shared with beneficiaries. First, an illustrated brochure was created in both Arabic and English and added to each test kit. The brochures explained the steps in performing the HIVST and how to interpret the result. In the leaflets, it was mentioned that any potential breach of confidentiality or forceful aspect or unwanted event should be reported to the NAP, similarly to the reporting path implemented since the beginning of VCT activities by NGOs.

To mobilize MSM and their partners, HIVST was promoted by the NAP through all its educational sessions, awareness campaigns and events such as World AIDS Day. On each occasion, the NAP included a short presentation about the importance of HIVST, its confidentiality, the instructions and the steps to perform it, and where and how to access it for free through a list of NGOs with their contacts and addresses. Moreover, the NAP made HIVST available for free at the NAP ART dispensing centre for PLHIV who wished to test their partner(s). Similarly, the NGOs used the same material to promote the HIVST through their on-site HCWs, social networking platforms (Facebook, Instagram, Twitter) and online dating applications (Grindr). Additionally, a hotline was set up at each NGO for beneficiaries to call about HIV counselling and guidance on testing.

While distributing the HIVSTs, NGOs anonymously collected data about each beneficiary’s age, consistency in condom use and HIV testing history. Additionally, each beneficiary was asked to call back and give the results of the HIVST to the NGO. The reporting tool was the same across all NGOs before and during COVID-19. HIVST data were reported to the NAP along with the standard reporting data on VCT services mentioned previously. Thus, HIV data reported to the NAP during the lockdown (mid-March to beginning of June 2020) only included HIVST data, with no VCT data, because there were no VCT activities. At the NAP, the data were combined, aggregated and analysed.

## Results

By the end of 2018, the NAP had procured and provided 1380 HIVST kits to the NGOs, and officially began collecting HIVST data in 2019. Out of the total 1380 HIVST kits (OraQuick HIV self-test; OraSure Technologies, Inc., Bethlehem, PA, USA) that NGOs received from the NAP, 1103 (79.9%) were distributed to MSM in 2019. The data collected at HIVST distribution (756/1103) showed that users/beneficiaries were on average 26.3 years old (range 18–57), 73.1% used condoms inconsistently and 26% had never had an HIV test.

Follow-up data were more challenging to collect. The data collected through follow-ups were impacted by a lack of beneficiary compliance to calling back and sharing their test results. Moreover, many beneficiaries distributed these tests to their peers or sex partners without any further contact with them, which impacted the data collected both at HIVST distribution and at the follow-up. Another challenge was that NGOs lacked the human and financial resources to actively follow up with their beneficiaries and thus relied on passive reporting of HIV results from the beneficiaries. This being said, 111 (10%) out of the 1103 beneficiaries who received an HIVST called back and notified the NGO about the HIV results. Among these, 2/111 users were reported HIV-positive. These cases were reported to the NAP and referred for confirmation and for treatment. They were enrolled into care at the NAP according to data from the NAP ART dispensing centre.

From January through May 2020, a total of 780 HIVST were provided to the NGOs, and 625 (80.1%) were distributed to beneficiaries. This period was divided into the period from January to mid-March (pre-COVID-19) and from mid-March to May (during COVID-19). Data collected during the distribution of HIVST from January to mid-March 2020 showed that beneficiaries were on average 29.1 years old, 71% of beneficiaries used condoms inconsistently and 21% of beneficiaries had never had an HIV test. However, compared to 2019, there was an improvement in the amount of follow-up data collected during COVID-19 (mid-March to May 2020) because of the absence of VCT services in the NGOs during COVID-19, shifting more efforts towards HIVST. In the COVID-19 era, HIVST allowed communities to experience partner-to-partner testing according to what was reported to the NGOs by beneficiaries. In the pre-COVID-19 period, a total of 79 out of 242 beneficiaries who received HIVST provided their HIV results. Among the 79 beneficiaries that reported the results of their HIV test, only one case was reported positive. In the period during COVID-19, a total of 370 beneficiaries out of 383 who received HIVST kits reported their HIV result to the NGOs, none of which was positive.

At the NAP and at NGOs, there have been no reports of unwanted incidents (no breach of confidentiality, no reported forceful testing or forceful notification of result) related to HIVST, which also includes the period during COVID-19.

## Discussion

The adoption of HIVST as a self-care intervention in Lebanon has been shown to be acceptable and feasible.

First, this self-care intervention was acceptable to local MSM who might face stigma in healthcare settings and by refugee MSM who lack access to healthcare. Self-care interventions such as HIVST are useful for all populations, and especially marginalized and vulnerable populations. Indeed, HIVST was found to decrease stigma about testing among MSM, as shown in a review of published data [12]. The fact that such high numbers of HIVST kits were distributed, and that beneficiaries reported their results (more importantly, during COVID-19), points towards a conclusion of general acceptance of the test by both native and refugee MSM. The distribution of HIVST for free had a positive impact, as otherwise the communities which were already socially (native MSM) or financially (refugee MSM) vulnerable would not have had any access to this diagnostic tool.

The acceptability of HIVST in Lebanon and other countries in the region was explored in two qualitative studies conducted in 2016. MSM (focus groups) from Tunisia, Morocco and Lebanon were interviewed about their acceptance and willingness to use HIVST [7, 8]. In both assessments, the participants acknowledged the potential for HIVST to contribute to a scale-up of testing but expressed concern about the accuracy of the test, self-harm and the absence of trained professional to interpret HIVST results. Access to HIVST via appropriate and safe delivery channels such as internet-based distributors, community centers, non-stigmatizing pharmacies, physician clinics, outreach workers or peers was acknowledged by participants as potential approaches for distribution. The direct assistance approach was preferred, which implies a major role of the HCWs in assisting the beneficiaries over the phone while they conduct their test. Our findings presented in the results highlight the major role of HCWs in HIVST implementation. Furthermore, with the lockdown having facilitated the follow-up on results, it is suggested that the HCW direct assistance approach is more productive: it allows the beneficiary to have a robust interpretation of his result and it limits lost or unreported cases. Thus, HIVST acceptability appears to be consistent in the previous WHO assessment [7, 8] and in our findings in the pre-COVID-19 era and during COVID-19.

Second, the implementation of HIVST was found to be feasible. Maatouk (2020) reviewed the published literature about the major steps to take before implementing HIVST including robust data collection, an update of testing guidelines, training of HCWs on guidelines, policies and regulations, and an update of HIV indicators to include HIVST-specific reporting tools [13]. These steps were adopted by the NAP to facilitate a more rapid scale-up of HIVST and updating of reporting and surveillance tools with HIVST. Moreover, the fear of safety regarding HIVST was obviated by the absence of any incident in 2 years.

Our second objective was to discuss how HIVST implementation has been impacted by COVID-19. There was increased demand for HIVST during COVID-19 because VCT services were completely paralysed due to the general lockdown and the stay-at-home curfews. The lockdown, curfews and the unavailability of VCT services encouraged individuals who wished to get tested for HIV to use HIVST. Moreover, the highly contagious nature of COVID-19 led to a drop in demand for VCT because individuals were experiencing increased stress and fear from the pandemic and were afraid to go to health facilities and hospitals. Many NGOs and other PHCs diverted their resources to COVID-19 response. The increased use of HIVST also allowed communities to experience partner-to-partner testing, which can strengthen trust between partners.

The responsibilities of HCWs at the NGOs shifted during COVID-19 towards promoting HIVST: HCWs were referring people more to HIVST and were encouraging people to use it. Consequently, people were using HIVST more, which enabled HCWs to be connected with beneficiaries and their HIV test results in different ways from before COVID-19. This shift in responsibilities allowed them to have more time for the follow-up with beneficiaries because they were unable to perform other duties such as VCT (370 beneficiaries were reached during COVID-19 versus 79 beneficiaries pre-COVID-19 in 2020). This increase in follow-up with beneficiaries was possible because the majority of NGOs had to reorganize their work and responsibilities during COVID-19 to alter how essential services could still be delivered, including HIV testing. In fact, the NGOs suffer from a scarcity of human and financial resources with the necessary skills to implement, supervise and support client counselling (HCW-assisted approach). Having such resources would help having a focal point HCW in each NGO who would be responsible for HIVST distribution, promotion, follow-up and data surveillance. Thus, the reorganization of care and redistribution of HCW duties within each NGO that occurred during COVID-19 needs to be maintained as an adaptation to improve HIVST implementation.

Despite the acceptability and feasibility of HIVST, however, there were several challenges faced during implementation. First, direct assistance with HIVST provided to beneficiaries by HCWs mitigates the fear of being alone during the stressful time of waiting for the test results. Second, some beneficiaries refused HIVST as a concept because of its long window (3 months between sexual exposure and ability to use HIVST) compared to the period for the conventional VCT (3 weeks between sexual exposure and ability to use VCT). These particular beneficiaries were advised to do the VCT instead. Third, some beneficiaries trusted the blood sample test over the saliva sample test used in HIVST. This challenge was solved by an explanation of the collection of antibodies through saliva to convince the beneficiaries of the solid scientific background of the test.

## Conclusion

HIVST implementation in Lebanon serves as an example of a community-led effort to introduce a self-care intervention in the MSM community. Despite the impact of COVID-19 on individuals and health systems, the scale-up and dissemination of HIVST was successful. In order to continue providing the same, or increased, level of HIVST services, NGOs will need to shift HCWs’ responsibilities and increase HIVST promotion. With continuous monitoring and evaluation of HIVST reported data, further implementation measures could lead to further expansion of HIVST services. Additionally, HIVST is not a solution to the stigma related to testing among MSM, which requires health equity and human rights laws in support of quality self-care interventions [10]. Continuing to target vulnerable communities, such as MSM, with HIVST will be important to reach those who are unable to access testing and other health services through traditional means.

## Data Availability

The datasets used and/or analysed during the current study are available from the corresponding author on reasonable request.

## Abbreviations

ART: Antiretroviral therapy
COVID-19: Coronavirus disease 2019
HCWs: Healthcare workers
HIVST: HIV self-testing
IEC: Information, education and communication
MSM: Men who have sex with men
NAP: National AIDS Program
NGOs: Nongovernmental organizations
PHCs: Primary healthcare centers
PLHIV: People living with HIV
UNAIDS: Joint United Nations Programme on HIV/AIDS
VCT: Voluntary counselling and testing
WHO: World Health Organization
